# Minimal effects from injunctive norm and contentiousness treatments on COVID-19 vaccine intentions: evidence from 3 countries

**DOI:** 10.1093/pnasnexus/pgac031

**Published:** 2022-05-13

**Authors:** John M Carey, Tracy Keirns, Peter John Loewen, Eric Merkley, Brendan Nyhan, Joseph B Phillips, Judy R Rees, Jason Reifler

**Affiliations:** Department of Government, Dartmouth College, Hanover, NH 03755, USA; UNH Survey Center, University of New Hampshire, Durham, NH 03823, USA; Department of Political Science, University of Toronto, Toronto, ONT M5S 3G3, Canada; Department of Political Science, University of Toronto, Toronto, ONT M5S 3G3, Canada; Department of Government, Dartmouth College, Hanover, NH 03755, USA; School of Psychology, University of Kent, Canterbury CT2 7NP, UK; Geisel School of Medicine, Dartmouth College, Hanover, NH 03755, USA; Department of Politics, University of Exeter, Amory Building, Exeter EX4 4RJ, UK

## Abstract

Does information about how other people feel about COVID-19 vaccination affect immunization intentions? We conducted preregistered survey experiments in Great Britain (5,456 respondents across 3 survey waves from September 2020 to February 2021), Canada (1,315 respondents in February 2021), and the state of New Hampshire in the United States (1,315 respondents in January 2021). The experiments examine the effects of providing accurate public opinion information to people about either public support for COVID-19 vaccination (an injunctive norm) or public beliefs that the issue is contentious. Across all 3 countries, exposure to this information had minimal effects on vaccination intentions even among people who previously held inaccurate beliefs about support for COVID-19 vaccination or its perceived contentiousness. These results suggest that providing information on public opinion about COVID vaccination has limited additional effect on people’s behavioral intentions when public discussion of vaccine uptake and intentions is highly salient.

Significance Statement Public health officials are currently struggling to determine which messages will most effectively promote vaccination as they seek to achieve the immunization rates required to end the COVID-19 pandemic. In this study, we focus on the potential of injunctive norm messages that describe which behaviors are seen as socially desirable. Our results indicate that providing accurate public opinion information about public support for people getting vaccinated has little measurable effect on reported vaccination intentions across Great Britain, Canada, and the United States. These results suggest that messages conveying widespread belief that people should get vaccinated are not effective in increasing intention to vaccinate—a highly relevant finding in countries where vaccinated majorities often convey disapproval of the unvaccinated.

## Introduction

Public health officials are currently struggling to determine which messages will most effectively promote vaccination as they seek to achieve the immunization rates required to end the COVID-19 pandemic (1). Though vaccines are safe, highly effective, and widely available, the rate of vaccination in countries where vaccines are widely available like the United States has slowed (2). Obstacles to vaccine uptake remain, including vaccine hesitancy (3), which is fueled by distrust of experts and false beliefs about the dangers of vaccination (4, 5). These challenges may be especially acute for COVID-19 due to the rapid pace at which vaccines were developed and deployed and the false or misleading messages about its safety and efficacy that have been promoted by some opinion leaders (6, 7).

The content of messaging about vaccines, and the social norms around their uptake, could affect willingness to vaccinate. Generally, people seek to engage in behaviors that others approve of (8, 9) and to avoid social sanction (10, 11), including on health behaviors such as exercise (12), cancer testing (13), and sunblock use (14). Although vaccine hesitancy is demonstrably difficult to reduce directly (15–17), norm-based messages could be effective because they can influence behaviors independently from attitudes (8, 9, 18).

This paper presents the results of a multicountry survey experiment testing the effects of 2 types of messages that could potentially affect vaccination intent: accurate public opinion information about how many people in one’s country want others to take the vaccine (an injunctive norm) or how many people perceive the issue as controversial. With the majority of the global population not yet vaccinated, our research adds to existing work that examines different routes for increasing COVID-19 vaccination intent. For example, Dai et al. (19) find that behavioral nudges sent by SMS can increase vaccination, and Ashworth et al. (20) find that personal health benefits messages seem to be particularly helpful.

We specifically examine messages that seek to communicate accurate public opinion information about how vaccination is perceived by other people, which could provide new insights into social influences on health behavior. In our study, we focus on the potential of injunctive norm messages to change intentions. These messages specifically emphasize whether or not behaviors are socially desirable, which may be a relevant consideration as people consider whether or not to get vaccinated. Injunctive norms are different from following how other people act in practice (21), which is a *descriptive norm*.

Specifically, people will generally seek to maximize how much they follow what others are doing (descriptive norms) and follow what others think they should do (injunctive norms). Descriptive norms are easy to follow as they only require mimicry of what others do. Injunctive norms require knowledge of what others want a person to do, but can be considerably more powerful through implicit social threat. Failure to follow injunctive norms comes with the expectation of social sanctions ranging from reprimands to ostracism (10). People are motivated to avoid exclusion because experiencing it even briefly can be psychologically painful (11).

In our study, we focus on the potential of injunctive norm messages. Milkman et al. (22) find a small positive effect of descriptive norm messages on flu vaccination intent in a large US study, while Ryoo and Kim (23) find that altering descriptive norm perceptions by making norm compliance or noncompliance visible affects vaccine hesitancy, especially when the norm is made salient. In contrast, Sinclair and Agerström (24) find only limited effects of descriptive norms on a sample of young adults in the UK. The effect of injunctive norm messaging may be greater. Both types of norms supply information on which behaviors are socially appropriate (25), but injunctive norms provide more direct information on which behaviors will elicit social sanctioning. Research on the efficacy of injunctive norms, however, is limited. Ryoo and Kim (23) find that their inducement of vaccine hesitancy through information on norm noncompliance can be eliminated by reminding people of the injunctive norm related to vaccination. Thaker and Ganchoudhuri (26) find an initial association between injunctive norm perceptions and COVID-19 vaccine intentions in cross-sectional panel survey data from New Zealand. We, therefore, preregistered the hypothesis that providing people with an injunctive norm message citing accurate public opinion data would increase their intent to vaccinate (**H1A**).

We further hypothesized that the effect of an injunctive norm messages would be greatest among respondents who previously underestimated the strength of the norm (**H1B**). Consistent with a Bayesian updating process, the effect of the new information provided by the public opinion data in the injunctive norm message should be greater as it increasingly differs from people’s prior beliefs. For instance, Ahler and Sood (27) find that the effect of correcting misperceptions about out-partisans is greatest for people whose prior beliefs were most inaccurate.

While prior research suggests that an injunctive norm message should increase uptake, our expectations about the effects of public opinion data about the perceived contentiousness of getting vaccinated are less clear. Controversy may signal that this vaccine is different from others or otherwise increase hesitancy (28, 29). Alternatively, however, discussion of the controversy over COVID-19 vaccination may remind people of the prevailing attitudes and behaviors expressed among the groups with which they identify, which could create null or even positive effects on net given that majorities favor vaccines in every country. Given these conflicting expectations, we identify the effects of exposure to information on perceived contention as a preregistered research question (**RQ1A**), including how it varies by people’s prior beliefs about contentiousness (**RQ1B**).

Finally, we consider potential spillover effects from correcting misperceptions on vaccine uptake. Our data from Great Britain and Canada are embedded in wider survey experiments that randomized exposure to fact-checks correcting several common misperceptions regarding COVID-19. (see Methods for details.) Correcting misperceptions may lead people to reconsider adjacent beliefs, including those about the safety of the COVID-19 vaccine. One possibility is that exposure to these fact-checks may make people more willing to reconsider other messages and information about COVID-19, which could enhance the effect of norm messaging. Alternatively, the fact-checks may persuade people independently of norm messaging, which could reduce the effect of norm messaging. We, therefore, examine preregistered research questions about the potential moderating effect of receiving fact-checks on the injunctive norm (**RQ2A**) and vaccine contention treatments (**RQ2B**).

Our findings indicate that providing accurate public opinion information about the (high) levels of support for other people getting vaccinated against COVID-19 has little measurable effect on people’s reported vaccination intentions. Providing public opinion data showing that COVID-19 vaccination is perceived as a contentious issue also has little measurable effect. These precisely estimated effects hold across samples in Great Britain, Canada, and the United States, and do not vary measurably by whether respondents underestimated the injunctive norm in favor of vaccination or by prior exposure to fact-checks debunking myths about COVID-19. These results suggest that messages conveying widespread belief that people should get vaccinated are not effective in increasing intention to vaccinate—a highly relevant finding in countries where vaccinated majorities often convey disapproval of those who have foregone immunization.

## Method

### Samples

We conducted surveys with respondents from Great Britain (England, Scotland, and Wales), Canada, and the state of New Hampshire in the United States. British data were obtained via a 3-wave panel study of respondents in England, Wales, and Scotland conducted by the online survey firm YouGov. The waves were conducted in 2020 September 11–29 (*n* = 5,456), 2020 December 10–23, and 2021 February 4–22. The Canadian survey consists of 1,315 respondents recruited in 2021 February 3–28 from Dynata’s online survey panel. This online nonprobability sample used quotas on region (i.e. Atlantic, Quebec, Ontario, and West) and language (i.e. French and English) along with interlocking quotas for education (i.e. degree and no degree), age (i.e. 18–34, 35–54, and 55 and older), and gender to match population benchmarks. The US data comes from 2,025 New Hampshire residents in the Granite State Poll online panel who were surveyed from 2021 January 21 to 25. This probability based online panel is representative of New Hampshire adults. Data were weighted by respondent sex, age, education, and region of the state to targets from the most recent American Community Survey (ACS) conducted by the US Census Bureau as well as party registration levels provided by the NH Secretary of State and 2020 election results in NH.

Table 1 reports pre- and post-treatment vaccine intention for respondents in all 3 countries as well as the rates at which British and Canadian respondents accurately estimated injunctive norms, and at which British respondents accurately estimated perceived vaccine contentiousness. The first survey wave in Great Britain was fielded before vaccine deployment (2020 September 11–29), so no respondents received it at baseline. By the time of the treatment wave (2021 February 4–22), 19% had done so. In Canada and NH (United States), pre- and post-treatment measures were collected in a single survey. Overall vaccine intentions were similar across countries, although NH (United States) respondents clustered more at the extremes of the scale.

**Table 1.** Descriptive statistics.

		Great Britain (%)	Canada (%)	NH (United States; %)
**Pretreatment vaccination intentions**	Already received	0	1	6
	Very likely/almost certain	43	48	57
	Somewhat likely/probably	13	17	12
	Slightly likely	13	13	–
	Not sure	–	–	3
	Slightly unlikely	7	6	
	Somewhat unlikely/probably not	5	4	5
	Very unlikely/almost certainly not	9	10	17
**Post-treatment vaccination intentions**	Already received	19	1	6
	Very likely	60	49	61
	Somewhat likely	8	16	9
	Slightly likely	5	13	3
	Slightly unlikely	2	5	2
	Somewhat unlikely	2	5	3
	Very unlikely	5	12	18
**Injunctive norm estimation**	Underestimated	21	24	–
	Accurate	62	37	–
	Overestimated	17	39	–
**Contention estimation**	Underestimated	40	–	–
	Accurate	60	–	–

Note: respondents are coded as underestimating (overestimating) an injunctive norm when their estimate of how many people in their country think others should get vaccines is at least 10% less (more) than the figure we estimated. Respondents are coded as underestimating contention when they strongly or somewhat disagreed or were not sure if whether to take an approved COVID-19 vaccine once eligible was a contentious topic in their country. Pretreatment vaccination intentions were measured in Wave 1 of the Great Britain survey (2020 September 11–29) and post-treatment intentions were measured in Wave 3 (2021 February 4–22). Pre- and post-treatment intentions were measured in the same wave as the experiment in the Canadian and NH (United States) samples.

Overall, respondents were reasonably accurate in their perceptions of the prevalence of the injunctive norm to vaccinate against COVID-19. British respondents estimated that 79% of their fellow citizens wanted others to get a vaccine, very close to the estimated figure from Wave 2 of the Great Britain survey (81%). Just under 2 out of 3 respondents (62%) were within 10 percentage points of this figure in either direction (71%–91%). In contrast, 21% of British respondents underestimated this figure by more than 10 percentage points, and a slightly smaller percentage (17%) overestimated this figure by more than 10 percentage points. On average, Canadian respondents estimated that 71% of their fellow citizens wanted others to get a vaccine, nearly identical to the estimated figure from another Dynata survey of Canadians (70%). That said, 39% overestimated this figure by 10 percentage points or more, and 24% underestimated it by the same amount.

### Materials and procedure

We provided respondents with accurate information about public beliefs related to COVID-19 vaccination. Respondents were randomly assigned to 1 of 3 conditions: an injunctive norm condition (with probability 40%), a vaccine contentiousness condition (with probability 30%), or a control condition with no additional information (with probability 30%). Though no formal a priori power analysis was conducted, we elected to deviate from random assignment with equal probability to increase our statistical power to test the effects of the injunctive norm treatment, which was our primary hypothesis of interest. (By contrast, the contentiousness treatment was a preregistered research question.)

In the injunctive norm condition, respondents were told that “a recent survey shows that [81% of Brits/70% of Canadians/64% of Americans] say people should get vaccinated with a COVID-19 vaccine once they are eligible.” In the vaccine contentiousness conditions, respondents were instead told that “a recent survey shows that [61% of Brits/64% of Canadians/61% of Americans] say COVID-19 vaccination is a contentious issue.” (The treatment did not specify which aspect(s) of COVID-19 vaccination was contentious, reflecting the original survey item, which was intended to capture the breadth of controversy around the issue. See Supplementary Material for all question wording.).

Each statistic provided to respondents in the injunctive norm and vaccine contentiousness conditions was the actual estimate from a recent survey conducted by the authors in the country in question. For Great Britain and Canada, the estimates were collected less than 2 months prior to data collection (the British data were collected in wave 2 of the 3-wave YouGov described above, and the Canadian data were collected in a separate Dynata survey conducted by the authors from 2020 December 15 to 2021 January 14). For New Hampshire, we provided respondents with national data collected from a representative sample of Americans in a YouGov study conducted less than 1 week prior to data collection (2021 January 15–18).

Because we used real-world data, treatment strength differs across countries for the injunctive norm message. The statistic provided to respondents varied from 64% among Americans to 81% of people in Great Britain (by contrast, results only varied slightly on contentiousness). This design choice was made for ethical reasons and to assess the effectiveness of injunctive norm information under real-world circumstances. We discuss the implications of this issue for our findings and future research further in the discussion section below.

Respondents in Great Britain were independently randomly assigned with equal probability to a fact-check condition or not in both Waves 2 and 3 (the wave in which the experiment reported in this paper was conducted). Respondents in Canada were randomly assigned with equal probability to a fact-check condition or not in 2 single-wave studies. These manipulations were orthogonal to the ones reported in this paper. For details on the wording and design of these manipulations, see the preregistrations for the studies in Great Britain (https://osf.io/bkfje/) and Canada (https://osf.io/jz86u/).

The key outcome measure is intent to vaccinate against COVID-19, which we measure on a 6-point Likert scale. Question wording varied slightly by country to reflect the context and timing of each survey. (In the United States, respondents were asked the following: “How likely is it that you will get a vaccine for the coronavirus once you are eligible?” In Canada, respondents were asked the following: “A vaccine for the coronavirus has been approved by Health Canada. How likely is it that you will choose to get an approved vaccine when you are eligible?” Finally, in Great Britain, respondents were asked the following: “How likely is it that you will get a vaccine for the coronavirus once you are eligible?” All could indicate they had already received the vaccine and otherwise responded on a 6-point Likert scale from “Very likely” to “Very unlikely.”) Treated respondents received injunctive norm or contentiousness information immediately prior to the vaccination intent question, whereas control condition respondents simply received the vaccination intent question.

In all of our datasets, some respondents reported having already received the vaccine at the time of our interview. Consistent with our preregistration, we treat respondents who report being vaccinated in 3 separate ways. In the main text, we report results in which we treat respondents who already received the vaccine as having equivalent vaccination intent to those who said they would “very likely” (Great Britain and Canada) or “almost certainly” (our US sample of New Hampshire residents) get vaccinated. The Supplementary Material reports results from Supplementary analyses in which those who reported being vaccinated are coded as having greater vaccination intent than those who were very likely/almost certainly getting the vaccine or as missing data. Our substantive results are robust to all 3 specifications.

To determine whether the effect of the treatment varied depending on the accuracy of people’s perceptions of the injunctive norm in favor of vaccination (a preregistered hypothesis), we administered a pretreatment question in the same wave as the experiment asking respondents in the British and Canadian samples to estimate the percentage of people in their country who say people should get a COVID-19 vaccine once they are eligible. We treat as “underestimators” those who underestimate the norm by 10 percentage points or more and “overestimators” as those who overestimate the norm by 10 percentage points or more. We conduct a similar analysis differentiating between people in Britain who see COVID-19 vaccines as a contentious issue (the majority) and those who do not (a preregistered research question). Due to an editing error, the text describing this specification was omitted from our preregistration, but the code and analysis largely mirrors the plan preregistered for a separate study (https://osf.io/wyb2e/?view_only = 2e53d08847ee4e59b6fb1fee5599d67c). We discuss this issue further in the Supplementary Material.

### Analytic strategy

All descriptive results reported below employ survey weights (i.e. probability weights) that are constructed to best approximate benchmarks from probability samples. By contrast, all experimental treatment effects are estimated without survey weights due to the loss of statistical power and additional assumptions that estimates of population average treatment effects require (30, 31). We test our primary hypotheses and research questions using ordinary least squares regression with HC2 robust standard errors. For covariate adjustment, we used a lasso variable selection procedure to determine the most prognostic covariates from a preregistered set to include in models for each dependent variable, which increases the precision of our estimates without appreciably increasing bias (32). Additional tests of covariate balance can be found in Tables S1–S3 (Supplementary Material). All deviations from our preregistration (https://osf.io/ebzad/?view_only = 34ed8d53da534284b0f63b941b02fdb6) are noted below (See Supplementary Material for details.). Models without covariates can be found in Tables S12–S19 (Supplementary Material).

### Results

**Fig. 1.**
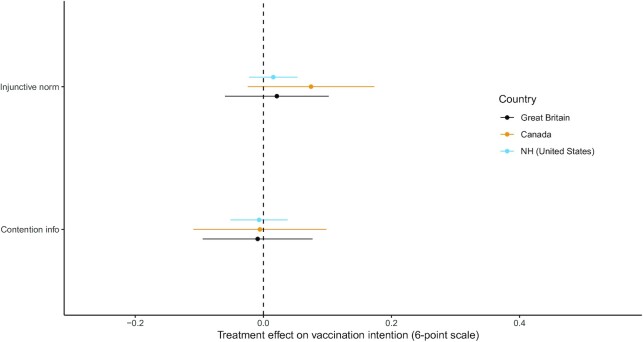
Treatment effect estimates on vaccine intention. Coefficients from OLS regressions of vaccine intention (6-point scale) on treatment assignment (see Tables S4–S6, Supplementary Material).

We hypothesized that respondents who were provided accurate information about the injunctive norm supporting COVID-19 vaccination would report higher intentions to get the vaccine relative to the control group (**H1A**). Treatment effects in each country by condition are depicted in Fig. 1.

Despite the differences in the injunctive norm statistics provided to respondents in the New Hampshire, Great Britain, and Canada, we find that the treatment had no measurable effect on our 6-point measure of vaccination intention in any of the 3 samples (see Tables S4–S6, Supplementary Material). These effects are precisely estimated in Great Britain and New Hampshire (95% CIs: Great Britain [−0.06, 0.10]; Canada [−0.01, 0.21]; NH (United States) [−0.02, 0.05]) and small in magnitude in all 3 samples (Great Britain: *d* = 0.02; Canada: *d* = 0.02.; NH (United States): *d* = 0.01). We also conducted an exploratory internal meta-analysis of all 3 samples. When we combine results from all 3 studies, the estimated effect of the injunctive norm treatment remains null and substantively small (β = 0.0227, 95% CIs [−0.0322, 0.0776]).

We also asked whether providing information about perceived levels of contention around COVID-19 vaccination affect immunization intentions (**RQ1A**). As Fig. 1 demonstrates, we find that the contention treatment (which was very similar across all 3 countries) had no measurable effect on vaccination intentions either. These effects are again precisely estimated (95% CIs: Great Britain [−0.09, 0.08]; Canada [−0.09, 0.14]; NH (United States) [−0.05, 0.04]; on a 6-point scale) and small in magnitude (Great Britain: *d* = −0.01, Canada: *d* = −0.00, NH (United States): *d* = −0.00). An internal meta-analysis finds the combined effect of the contention treatment is negative and statistically distinguishable from zero, but incredibly small (β = −0.0069, 95% CIs [−0.0101, −0.0038]).

**Fig. 2.**
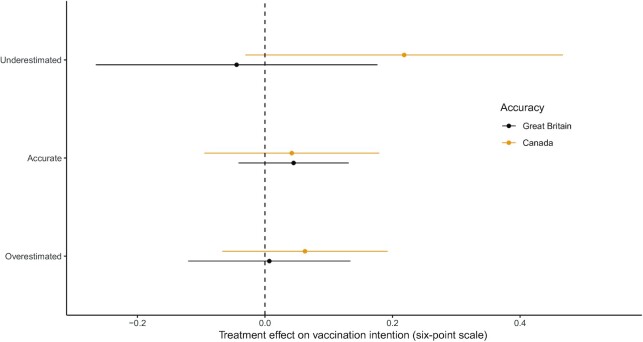
Treatment effect estimates by accuracy of prior beliefs about injunctive norm. Effect estimates from OLS models of vaccine intention interacting treatment assignment with indicators for accuracy of prior beliefs about injunctive norm in favor of COVID-19 vaccination (see Tables S15 and S16, Supplementary Material).

We further hypothesized that the effect of the injunctive norm treatment on vaccine intentions would be greater (more positive) among respondents who previously underestimated the injunctive norm around vaccination (**H1B**). We tested this hypothesis in our British and Canadian samples (the New Hampshire sample from the United States did not include a prior estimate of injunctive norm). Treatment effects are depicted in Fig. 2.

We do not find support for H1B. The effect of the injunctive norm treatment does not measurably vary by the accuracy of respondent’s prior estimates of the injunctive norm supporting people in their country getting vaccinated in either the British or Canadian sample (see Tables S7 and S8, Supplementary Material). We also find in an exploratory analysis that the effect of the injunctive norm treatment does not vary by prior perceptions of injunctive norms on the original 0–100 scale (see Figure S1, Supplementary Material).

**Fig. 3.**
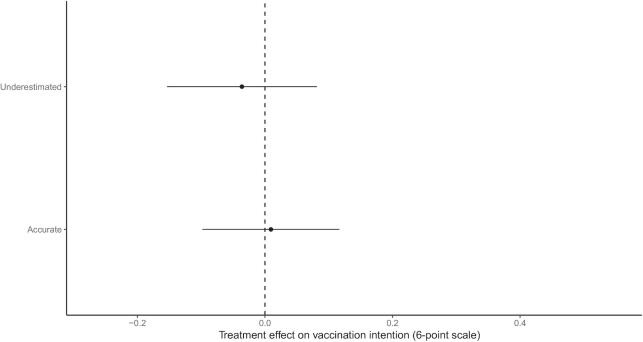
Treatment effect estimates by accuracy of prior beliefs about contention (Great Britain). Effect estimates from OLS models of vaccine intention interacting treatment assignment with indicators for accuracy of prior beliefs about perceived contentiousness of COVID-19 vaccination (see Table S9, Supplementary Material).

Similarly, we asked whether the effect of providing accurate information about the perceived contentiousness of COVID-19 vaccination varies with the accuracy of people’s beliefs about levels of contention (**RQ1B**). We tested this research question in our British sample exclusively. Treatment effects can be found in Fig. 3. We find that regardless of prior perceived contention surrounding COVID vaccination uptake, being alerted to this contention had no measurable effect on vaccination intentions.

Finally, we asked whether receiving a fact-check about COVID-19 misperceptions prior to treatment moderated the effects of the injunctive norm (**RQ2A**) or contention (**RQ2B**) treatments on vaccination intentions. In the British and Canadian samples, we estimated models interacting the injunctive norm treatment with whether or not respondents received a fact-check treatment (along with the appropriate constituent terms). We then fail to reject the joint null hypothesis test that all constituent and interactive terms containing the fact-check treatments are 0 in both cases (see Tables S10 and S11, Supplementary Material).

## Discussion

An analysis of 6,530 adults in 3 countries shows that providing public opinion information about the injunctive norm supporting COVID vaccination has minimal effect on intended vaccine uptake even among people who had previously underestimated support for the norm. We similarly find that survey data reminding people of the perceived level of contentiousness around COVID vaccination has negligible effects on vaccine intention. These results suggest that messages providing accurate information about public beliefs related to vaccination have little effect on vaccination intentions.

Our findings contrast somewhat with prior research showing positive effects of descriptive norms on COVID-19 vaccination intention (22,23), though other findings are more mixed (24). Importantly, though, (22) find weak effects in the United States, much like we see here. Future work exploring the reasons behind cross-national variation in the efficacy of norm-based messages—descriptive or injuctive—is, thus vitally important. Injunctive norms might have greater potential to affect behavior because of the potential for social sanction (25), yet our findings show no indication that exposure to accurate public opinion information increased vaccination intention in any country, including ones in which the consensus that other people should get vaccinated was especially strong. These results provide a stronger evidentiary basis for assessing the effects of injunctive norms on COVID-19 vaccination intention than the correlational results reported by Thaker and Ganchoudhuri (26) using data from New Zealand.

One possible explanation for this finding is that many respondents had relatively accurate perceptions of injunctive norms prior to the study, limiting the potential impact of the treatment. However, we found no evidence of heterogeneous treatment effects by whether people over or underestimated the strength of injunctive norms. Scholars should examine the role of pretreatment accuracy of norm perceptions as a moderating factor in experiments of this type.

These findings have several other limitations that should also be addressed in future research. First, our experiments were conducted at a particular point in the course of the COVID-19 pandemic in our 3 survey countries. Results may vary as conditions change or public opinion shifts. Second, our outcome variable consists of a single survey item, which may result in greater measurement error than a multi-item scale. Third, replication with a national sample in the United States and with a nationally representative sample in Canada would be desirable. Fourth, people who overestimate or underestimate various norms may differ from those who accurately perceive them on other dimensions; these potential moderators are not randomly assigned. Fifth, our treatment strength varied by country for the injunctive norm treatment, though we observed no corresponding evidence of country-level heterogeneity in our results. Finally, it is important to acknowledge that some respondents may already have been influenced by social norms related to COVID-19 vaccination or received similar information to the treatments before they participated in our study (33).

Nonetheless, these results provide valuable insight into both the effects of information about public opinion on COVID-19 vaccination on immunization intentions and the effect of injunctive norms on behavioral intentions more generally.

## Supplementary Material

pgac031_Supplemental_Files

## Data Availability

All data and analyses can be found in a Harvard DataVerse repository at https://doi.org/10.7910/DVN/TRFWBI.
