# Correction to: Minimal effects from injunctive norm and contentiousness treatments on COVID-19 vaccine intentions: evidence from 3 countries

**DOI:** 10.1093/pnasnexus/pgad458

**Published:** 2024-01-30

This is a correction to: John M Carey, Tracy Keirns, Peter John Loewen, Eric Merkley, Brendan Nyhan, Joseph B Phillips, Judy R Rees, Jason Reifler, Minimal effects from injunctive norm and contentiousness treatments on COVID-19 vaccine intentions: evidence from 3 countries, *PNAS Nexus*, Volume 1, Issue 2, May 2022, pgac031, https://doi.org/10.1093/pnasnexus/pgac031

During a retroactive audit conducted by *PNAS Nexus*, it was discovered that this paper was missing a statement acknowledging compliance with the *PNAS Nexus* Human and Animal Participants and Clinical Trials policy:

The study was approved by the Dartmouth IRB (CPHS#STUDY00032068), University of New Hampshire Research Integrity Services (#IRB-FY2021-53), University of Toronto Social Sciences, Humanities and Education Research Ethics Board (HPR-00025662), and the University of Exeter SSIS Ethics Committee (#201920-148). All participants gave informed consent.

This error has been corrected in the original article.

