# Supplementary material for: Minimal effects from injunctive norm and contentiousness treatments on COVID-19 vaccine intentions: evidence from 3 countries
Source: PNAS Nexus. 2022 May 13;1(2):pgac031. doi: 10.1093/pnasnexus/pgac031 (PMC9802041; doi:10.1093/pnasnexus/pgac031)
Supplement: pgac031_Supplemental_Files [file pgac031_supplemental_files.zip › PNASNEXUS-PNASNEXUS-2021-00156-s02.pdf]

Minimal effects from injunctive norm and contentiousness treatments on COVID-19 vaccine intentions: Evidence from three countries

John Carey[1], Tracy Keirns[2], Peter John Loewen[3], Eric Merkley[3], Brendan Nyhan[1], Joseph B. Phillips[4], Judy R. Rees[5], and Jason Reifler[†6]

[1]Department of Government, Dartmouth College, Hanover, NH, USA
[2]UNH Survey Center, University of New Hampshire, Durham, NH, USA
[3]Department of Political Science, University of Toronto, Toronto, ONT, Canada
[4]School of Psychology, University of Kent, Canterbury, United Kingdom
[5]Geisel School of Medicine, Dartmouth College, Hanover, NH, USA
[6]Department of Politics, University of Exeter, Exeter, United Kingdom

[†]Corresponding author: J.Reifler@exeter.ac.uk. University of Exeter, Amory Building, Exeter, EX4 4RJ, United Kingdom.

Abstract

Does information about how other people feel about COVID-19 vaccination affect immunization intentions? We conducted pre-registered survey experiments in Great Britain (5,456 respondents across three survey waves from September 2020 - February 2021), Canada (1,315 respondents in February 2021), and the state of New Hampshire in the United States (1,315 respondents in January 2021). The experiments examine the effects of providing accurate public opinion information to people about either public support for COVID-19 vaccination (an injunctive norm) or public beliefs that the issue is contentious. Across all three countries, exposure to this information had minimal effects on vaccination intentions even among people who previously held inaccurate beliefs about support for COVID-19 vaccination or its perceived contentiousness. These results suggest that providing information on public opinion about COVID vaccination has limited additional effect on people's behavioral intentions when public discussion of vaccine uptake and intentions is highly salient.

Significance statement

Public health officials are currently struggling to determine which messages will most effectively promote vaccination as they seek to achieve the immunization rates required to end the COVID-19 pandemic. In this study, we focus on the potential of injunctive norm messages that describe which behaviors are seen as socially desirable. Our results indicate that providing accurate public opinion information about public support for people getting vaccinated has little measurable effect on reported vaccination intentions across Great Britain, Canada, and the United States. These results suggest that messages conveying widespread belief that people should get vaccinated are not effective in increasing intention to vaccinate — a highly relevant finding in countries where vaccinated majorities often convey disapproval of the unvaccinated.

Public health officials are currently struggling to determine which messages will most effectively promote vaccination as they seek to achieve the immunization rates required to end the COVID-19 pandemic (Bartsch et al. 2020). Though vaccines are safe, highly effective, and widely available, the rate of vaccination in countries where vaccines are widely available like the United States has slowed (Glenza 2021). Obstacles to vaccine uptake remain, including vaccine hesitancy {(Gravelle et al. In press), which is fueled by distrust of experts and false beliefs about the dangers of vaccination (Hornsey, Lobera and Díaz-Catalán 2020; Lee et al. 2016). These challenges may be especially acute for COVID-19 due to the rapid pace at which vaccines were developed and deployed and the false or misleading messages about its safety and efficacy that have been promoted by some opinion leaders (Callaghan et al. 2021; Ledford, Cyranoski and Van Noorden 2020).

The content of messaging about vaccines, and the social norms around their uptake, could affect willingness to vaccinate. Generally, people seek to engage in behaviors that others approve of (Christensen et al. 2004; Cialdini, Kallgren and Reno 1991) and to avoid social sanction (Reno, Cialdini and Kallgren 1993; MacDonald and Leary 2005), including on health behaviors such as exercise (Wally and Cameron 2017), cancer testing (Smith-McLallen and Fishbein 2008), and sunblock use (Reid and Aiken 2013). Although vaccine hesitancy is demonstrably difficult to reduce directly (Dubé et al. 2015; Jarrett et al. 2015; Nyhan et al. 2014), norm-based messages could be effective because they can influence behaviors independently from attitudes (Christensen et al. 2004; Cialdini, Kallgren and Reno 1991; Paluck 2009).

This paper presents the results of a multi-country survey experiment testing the effects of two types of messages that could potentially affect vaccination intent: accurate public opinion information about how many people in one's country want others to take the vaccine (an injunctive norm) or how many people perceive the issue as controversial. With the majority of the global population not yet vaccinated, our research adds to existing work that examines different routes for increasing COVID-19 vaccination intent. For example, Dai et al. (2021) find that behavioral nudges sent by SMS can increase vaccination and Ashworth et al. (2021) find that personal health benefits messages seem to be particularly helpful.

We specifically examine messages that seek to communicate accurate public opinion information about how vaccination is perceived by other people, which could provide new insights into social influences on health behavior. In our study, we focus on the potential of injunctive norm messages to change intentions. These messages specifically emphasize whether or not behaviors are socially desirable, which may be a relevant consideration as people consider whether or not to get vaccinated. Injunctive norms are different from following how other people act in practice (Cialdini 2007), which is a *descriptive norm*.

Specifically, people will generally seek to maximize how much they follow what others are doing (descriptive norms) and follow what others think they should do (injunctive norms). Descriptive norms are easy to follow as they only require mimicry of what others do. Injunctive norms require knowledge of what others want a person to do, but can be considerably more powerful through implicit social threat. Failure to follow injunctive norms comes with the expectation of social sanctions ranging from reprimands to ostracism (Reno, Cialdini and Kallgren 1993). People are motivated to avoid exclusion because experiencing it even briefly can be psychologically painful (MacDonald and Leary 2005).

In our study, we focus on the potential of injunctive norm messages. Milkman et al. (N.d.) find a small positive effect of descriptive norm messages on flu vaccination intent in a large US study, while Ryoo and Kim (2021) find that altering descriptive norm perceptions by making norm compliance or non-compliance visible affects vaccine hesitancy, especially when the norm is made salient. In contrast, Sinclair and Agerström (2021) find only limited effects of descriptive norms on a sample of young adults in the UK. The effect of injunctive norm messaging may be greater. Both types of norms supply information on which behaviors are socially appropriate (Schultz et al. 2007), but injunctive norms provide more direct information on which behaviors will elicit social sanctioning. Research on the efficacy of injunctive norms, however, is limited. Ryoo and Kim (2021) find that their inducement of vaccine hesitancy through information on norm non-compliance can be eliminated by reminding people of the injunctive norm related to vaccination. Thaker and Ganchoudhuri (2021) find an initial association between injunctive norm perceptions and COVID-19

vaccine intentions in cross-sectional panel survey data from New Zealand. We therefore preregistered the hypothesis that providing people with an injunctive norm message citing accurate public opinion data would increase their intent to vaccinate (**H1A**).

We further hypothesized that the effect of an injunctive norm messages would be greatest among respondents who previously underestimated the strength of the norm (**H1B**). Consistent with a Bayesian updating process, the effect of the new information provided by the public opinion data in the injunctive norm message should be greater as it increasingly differs from people's prior beliefs. For instance, Ahler and Sood (2018) find that the effect of correcting misperceptions about out-partisans is greatest for people whose prior beliefs were most inaccurate.

While prior research suggests that an injunctive norm message should increase uptake, our expectations about the effects of public opinion data about the perceived contentiousness of getting vaccinated are less clear. Controversy may signal that this vaccine is different from others or otherwise increase hesitancy (Fowler et al. 2012; Fowler and Gollust 2015). Alternatively, however, discussion of the controversy over COVID-19 vaccination may remind people of the prevailing attitudes and behaviors expressed among the groups with which they identify, which could create null or even positive effects on net given that majorities favor vaccines in every country. Given these conflicting expectations, we identify the effects of exposure to information on perceived contention as a preregistered research question (**RQ1A**), including how it varies by people's prior beliefs about contentiousness (**RQ1B**).

Finally, we consider potential spillover effects from correcting misperceptions on vaccine uptake. Our data from Great Britain and Canada are embedded in wider survey experiments that randomized exposure to fact-checks correcting several common misperceptions regarding COVID-19. (See Methods for details.) Correcting misperceptions may lead people to reconsider adjacent beliefs, including those about the safety of the COVID-19 vaccine. One possibility is that exposure to these fact-checks may make people more willing to reconsider other messages and information about COVID-19, which could enhance the effect of norm messaging. Alternatively, the fact-checks may persuade people independently of norm messaging, which could reduce the effect of norm

messaging. We therefore examine preregistered research questions about the potential moderating effect of receiving fact-checks on the injunctive norm (**RQ2A**) and vaccine contention treatments (**RQ2B**).

Our findings indicate that providing accurate public opinion information about the (high) levels of support for other people getting vaccinated against COVID-19 has little measurable effect on people's reported vaccination intentions. Providing public opinion data showing that COVID-19 vaccination is perceived as a contentious issue also has little measurable effect. These precisely estimated effects hold across samples in GB, Canada, and the US and do not vary measurably by whether respondents underestimated the injunctive norm in favor of vaccination or by prior exposure to fact-checks debunking myths about COVID-19. These results suggest that messages conveying widespread belief that people should get vaccinated are not effective in increasing intention to vaccinate — a highly relevant finding in countries where vaccinated majorities often convey disapproval of those who have foregone immunization.

Method

Samples

We conducted surveys with respondents from Great Britain (England, Scotland, and Wales), Canada, and the state of New Hampshire in the United States. British data were obtained via a three-wave panel study of respondents in England, Wales, and Scotland conducted by the online survey firm YouGov. The waves were conducted September 11–29, 2020 (n=5,456), December 10–23, 2020, and February 4–22, 2021. The Canadian survey consists of 1,315 respondents recruited February 3–28, 2021 from Dynata's online survey panel. This online non-probability sample used quotas on region (i.e., Atlantic, Quebec, Ontario, West) and language (i.e., French, English) along with interlocking quotas for education (i.e., degree, no degree), age (i.e., 18-34, 35-54, 55 and older), and gender to match population benchmarks. The U.S. data comes from 2,025 New Hampshire residents in the Granite State Poll online panel who were surveyed from January 21–25, 2021. This

probability based online panel is representative of New Hampshire adults. Data were weighted by respondent sex, age, education, and region of the state to targets from the most recent American Community Survey (ACS) conducted by the U.S. Census Bureau as well as party registration levels provided by the NH Secretary of State and 2020 election results in NH.

[Table 1 about here]

Table 1 reports pre- and post-treatment vaccine intention for respondents in all three countries as well as the rates at which British and Canadian respondents accurately estimated injunctive norms and at which British respondents accurately estimated perceived vaccine contentiousness. The first survey wave in Great Britain was fielded before vaccine deployment (September 11–29, 2020), so no respondents received it at baseline. By the time of the treatment wave (February 4–22, 2021), 19% had done so. In Canada and NH (US), pre- and post-treatment measures were collected in a single survey. Overall vaccine intentions were similar across countries, although NH (US) respondents clustered more at the extremes of the scale.

Overall, respondents were reasonably accurate in their perceptions of the prevalence of the injunctive norm to vaccinate against COVID-19. British respondents estimated that 79% of their fellow citizens wanted others to get a vaccine, very close to the estimated figure from Wave 2 of the GB survey (81%). Just under 2 out of 3 respondents (62%) were within 10 percentage points of this figure in either direction (71–91%). In contrast, 21% of British respondents underestimated this figure by more than 10 percentage points, and a slightly smaller percentage (17%) overestimated this figure by more than 10 percentage points. On average, Canadian respondents estimated that 71% of their fellow citizens wanted others to get a vaccine, nearly identical to the estimated figure from another Dynata survey of Canadians (70%). That said, 39% overestimated this figure by 10 percentage points or more, and 24% underestimated it by the same amount.

Materials and procedure

We provided respondents with accurate information about public beliefs related to COVID-19 vaccination. Respondents were randomly assigned to one of three conditions: an injunctive norm condition (with probability 40%), a vaccine contentiousness condition (with probability 30%), or a control condition with no additional information (with probability 30%). Though no formal a priori power analysis was conducted, we elected to deviate from random assignment with equal probability to increase our statistical power to test the effects of the injunctive norm treatment, which was our primary hypothesis of interest. (By contrast, the contentiousness treatment was a preregistered research question.)

In the injunctive norm condition, respondents were told that "a recent survey shows that [81% of Brits/70% of Canadians/64% of Americans] say people should get vaccinated with a COVID-19 vaccine once they are eligible." In the vaccine contentiousness conditions, respondents were instead told that "a recent survey shows that [61% of Brits/64% of Canadians/61% of Americans] say COVID-19 vaccination is a contentious issue." (The treatment did not specify which aspect(s) of COVID-19 vaccination was contentious, reflecting the original survey item, which was intended to capture the breadth of controversy around the issue. See Supplementary Information (SI) for all question wording.)

Each statistic provided to respondents in the injunctive norm and vaccine contentiousness conditions was the actual estimate from a recent survey conducted by the authors in the country in question. For Great Britain and Canada, the estimates were collected less than two months prior to data collection (the British data were collected in wave 2 of the three-wave YouGov described above and the Canadian data were collected in a separate Dynata survey conducted by the authors from December 15, 2020–January 14, 2021). For New Hampshire, we provided respondents with national data collected from a representative sample of Americans in a YouGov study conducted less than one week prior to data collection (January 15–18, 2021).

Because we used real-world data, treatment strength differs across countries for the injunctive norm message. The statistic provided to respondents varied from 64% among Americans to 81%

of people in Great Britain (by contrast, results only varied slightly on contentiousness). This design choice was made for ethical reasons and to assess the effectiveness of injunctive norm information under real-world circumstances. We discuss the implications of this issue for our findings and future research further in the discussion section below.

Respondents in Great Britain were independently randomly assigned with equal probability to a fact-check condition or not in both Wave 2 and Wave 3 (the wave in which the experiment reported in this paper was conducted). Respondents in Canada were randomly assigned with equal probability to a fact-check condition or not in two single-wave studies. These manipulations were orthogonal to the ones reported in this paper. For details on the wording and design of these manipulations, see the preregistrations for the studies in Great Britain (`https://osf.io/bkfje/`) and Canada (`https://osf.io/jz86u/`).

The key outcome measure is intent to vaccinate against COVID-19, which we measure on a six-point Likert scale. Question wording varied slightly by country to reflect the context and timing of each survey. (In the US, respondents were asked the following: "How likely is it that you will get a vaccine for the coronavirus once you are eligible?" In Canada, respondents were asked the following: "A vaccine for the coronavirus has been approved by Health Canada. How likely is it that you will choose to get an approved vaccine when you are eligible?" Finally, in Great Britain, respondents were asked the following: "How likely is it that you will get a vaccine for the coronavirus once you are eligible?" All could indicate they had already received the vaccine and otherwise responded on a six-point Likert scale from "Very likely" to "Very unlikely.") Treated respondents received injunctive norm or contentiousness information immediately prior to the vaccination intent question, whereas control condition respondents simply received the vaccination intent question.

In all of our datasets, some respondents reported having already received the vaccine at the time of our interview. Consistent with our preregistration, we treat respondents who report being vaccinated in three separate ways. In the main text, we report results in which we treat respondents who already received the vaccine as having equivalent vaccination intent to those who said they would "very likely" (Great Britain and Canada) or "almost certainly" (our US sample of New Hampshire

residents) get vaccinated. The SI reports results from Supplementary analyses in which those who reported being vaccinated are coded as having greater vaccination intent than those who were very likely/almost certainly getting the vaccine and as missing data. Our substantive results are robust to all three specifications.

To determine whether the effect of the treatment varied depending on the accuracy of people's perceptions of the injunctive norm in favor of vaccination (a preregistered hypothesis), we administered a pre-treatment question in the same wave as the experiment asking respondents in the British and Canadian samples to estimate the percentage of people in their country who say people should get a COVID-19 vaccine once they are eligible. We treat as "underestimators" those who underestimate the norm by 10 percentage points or more and "overestimators" as those who overestimate the norm by 10 percentage points or more. We conduct a similar analysis differentiating between people in Britain who see COVID-19 vaccines as a contentious issue (the majority) and those who do not (a preregistered research question). Due to an editing error, the text describing this specification was omitted from our preregistration, but the code and analysis largely mirrors the plan preregistered for a separate study (`https://osf.io/wyb2e/?view_only=2e53d08847ee4e59b6fb1fee5599d67c`). We discuss this issue further in the Supplementary Information.

Analytic strategy

All descriptive results reported below employ survey weights (i.e., probability weights) that are constructed to best approximate benchmarks from probability samples. By contrast, all experimental treatment effects are estimated without survey weights due to the loss of statistical power and additional assumptions that estimates of population average treatment effects require (Franco et al. 2017; Miratrix et al. 2018). We test our primary hypotheses and research questions using ordinary least squares regression with HC2 robust standard errors. For covariate adjustment, we used a lasso variable selection procedure to determine the most prognostic covariates from a preregistered set to include in models for each dependent variable, which increases the precision of our

estimates without appreciably increasing bias (Bloniarz et al. 2016). Additional tests of covariate balance can be found in Tables S1-S3 in the SI. All deviations from our preregistration (`https://osf.io/ebzad/?view_only=34ed8d53da534284b0f63b941b02fdb6`) are noted below. (See SI for details.) Models without covariates can be found in Tables S12-S19 in the SI.

Results

We hypothesized that respondents who were provided accurate information about the injunctive norm supporting COVID-19 vaccination would report higher intentions to get the vaccine relative to the control group (**H1A**). Treatment effects in each country by condition are depicted in Figure 1.

Figure 1: Treatment effect estimates on vaccine intention.

Coefficients from OLS regressions of vaccine intention (six-point scale) on treatment assignment (see Tables S4–S6 in the Supplementary Information).

Despite the differences in the injunctive norm statistics provided to respondents in the New Hampshire, Great Britain, and Canada, we find that the treatment had no measurable effect on our six-point measure of vaccination intention in any of the three samples (see Tables S4–S6 in the SI). These effects are precisely estimated in Great Britain and New Hampshire (95% CIs: GB [-0.06, 0.10]; Canada [-0.01, 0.21]; NH (US) [-0.02, 0.05]) and small in magnitude in all three samples (GB: d=.02; Canada: d=.02.; NH (US): d=.01). We also conducted an exploratory internal meta-analysis of all three samples. When we combine results from all three studies, the estimated effect of the injunctive norm treatment remains null and substantively small (β=0.0227, 95% CIs [-0.0322, 0.0776]).

We also asked whether providing information about perceived levels of contention around COVID-19 vaccination affect immunization intentions (**RQ1A**). As Figure 1 demonstrates, we find that the contention treatment (which was very similar across all three countries) had no measurable effect on vaccination intentions either. These effects are again precisely estimated (95% CIs: GB [-0.09,

0.08]; Canada [-0.09, 0.14]; NH (US) [-0.05, 0.04]; on a 6-point scale) and small in magnitude (GB: d=-.01, Canada: d=-.00, NH (US): d=-.00). An internal meta-analysis finds the combined effect of the contention treatment is negative and statistically distinguishable from zero, but incredibly small (β=-0.0069, 95% CIs [-0.0101, -0.0038]).

We further hypothesized that the effect of the injunctive norm treatment on vaccine intentions would be greater (more positive) among respondents who previously underestimated the injunctive norm around vaccination (**H1B**). We tested this hypothesis in our British and Canadian samples (the New Hampshire sample from the United States did not include a prior estimate of injunctive norm). Treatment effects are depicted in Figure 2.

Figure 2: Treatment effect estimates by accuracy of prior beliefs about injunctive norm

Effect estimates from OLS models of vaccine intention interacting treatment assignment with indicators for accuracy of prior beliefs about injunctive norm in favor of COVID-19 vaccination (see Tables S15 and S16 in the SI).

We do not find support for H1B. The effect of the injunctive norm treatment does not measurably vary by the accuracy of respondent's prior estimates of the injunctive norm supporting people in their country getting vaccinated in either the British or Canadian sample (see Tables S7 and S8 in the SI). We also find in an exploratory analysis that the effect of the injunctive norm treatment does not vary by prior perceptions of injunctive norms on the original 0–100 scale (see Figure S1 in the SI).

Figure 3: Treatment effect estimates by accuracy of prior beliefs about contention (GB)

Effect estimates from OLS models of vaccine intention interacting treatment assignment with indicators for accuracy of prior beliefs about perceived contentiousness of COVID-19 vaccination (see Table S9 in the Supplementary Information).

Similarly, we asked whether the effect of providing accurate information about the perceived contentiousness of COVID-19 vaccination varies with the accuracy of people's beliefs about levels of contention (**RQ1B**). We tested this research question in our British sample exclusively. Treatment

effects can be found in Figure 3. We find that regardless of prior perceived contention surrounding COVID vaccination uptake, being alerted to this contention had no measurable effect on vaccination intentions.

Finally, we asked whether receiving a fact-check about COVID-19 misperceptions prior to treatment moderated the effects of the injunctive norm (**RQ2A**) or contention (**RQ2B**) treatments on vaccination intentions. In the British and Canadian samples, we estimated models interacting the injunctive norm treatment with whether or not respondents received a fact-check treatment (along with the appropriate constituent terms). We then fail to reject the joint null hypothesis test that all constituent and interactive terms containing the fact-check treatments are zero in both cases (see Tables S10 and S11 in the SI).

Discussion

An analysis of 6,530 adults in three countries shows that providing public opinion information about the injunctive norm supporting COVID vaccination has minimal effect on intended vaccine uptake even among people who had previously underestimated support for the norm. We similarly find that survey data reminding people of the perceived level of contentiousness around COVID vaccination has negligible effects on vaccine intention. These results suggest that messages providing accurate information about public beliefs related to vaccination have little effect on vaccination intentions.

Our findings contrast somewhat with prior research showing positive effects of descriptive norms on COVID-19 vaccination intention (Milkman et al. N.d.; Ryoo and Kim 2021), though other findings are more mixed (Sinclair and Agerström 2021). Importantly, though, (Milkman et al. N.d.) find weak effects in the US, much like we see here. Future work exploring the reasons behind cross-national variation in the efficacy of norm-based messages – descriptive or injuctive – is thus vitally important. Injunctive norms might have greater potential to affect behavior because of the potential for social sanction (Schultz et al. 2007), yet our findings show no indication that exposure to accurate public opinion information increased vaccination intention in any country, including

ones in which the consensus that other people should get vaccinated was especially strong. These results provide a stronger evidentiary basis for assessing the effects of injunctive norms on COVID-19 vaccination intention than the correlational results reported by Thaker and Ganchoudhuri (2021) using data from New Zealand.

One possible explanation for this finding is that many respondents had relatively accurate perceptions of injunctive norms prior to the study, limiting the potential impact of the treatment. However, we found no evidence of heterogeneous treatment effects by whether people over- or underestimated the strength of injunctive norms. Scholars should examine the role of pre-treatment accuracy of norm perceptions as a moderating factor in experiments of this type.

These findings have several other limitations that should also be addressed in future research. First, our experiments were conducted at a particular point in the course of the COVID-19 pandemic in our three survey countries. Results may vary as conditions change or public opinion shifts. Second, our outcome variable consists of a single survey item, which may result in greater measurement error than a multi-item scale. Third, replication with a national sample in the US and with a nationally representative sample in Canada would be desirable. Fourth, people who overestimate or underestimate various norms may differ from those who accurately perceive them on other dimensions; these potential moderators are not randomly assigned. Fifth, our treatment strength varied by country for the injunctive norm treatment, though we observed no corresponding evidence of country-level heterogeneity in our results. Finally, it is important to acknowledge that some respondents may already have been influenced by social norms related to COVID-19 vaccination or received similar information to the treatments before they participated in our study (Druckman and Leeper 2012).

Nonetheless, these results provide valuable insight into both the effects of information about public opinion on COVID-19 vaccination on immunization intentions and the effect of injunctive norms on behavioral intentions more generally.

Acknowledgements

We thank the National Science Foundation (grant number 2028485), the Economic and Social Research Council (grant number ES/V004883/1), the Munk School of Global Affairs & Public Policy and the Dean of the Faculty of Arts & Science at the University of Toronto, the Hitchcock Foundation, the Jack and Dorothy Byrne Foundation, and Dartmouth College COVID-19 Spark Funds for funding support. All conclusions and any errors are our own.

Competing Interest Statement

The authors declare no competing interest.

Data Availability

All data and analyses can be found in a Harvard DataVerse repository at https://doi.org/10.7910/DVN/TRFWBI.

References

Ahler, Douglas J and Gaurav Sood. 2018. "The parties in our heads: Misperceptions about party composition and their consequences." The Journal of Politics 80(3):964–981.

Ashworth, Madison, Linda Thunström, Todd L Cherry, Stephen C Newbold and David C Finnoff. 2021. "Emphasize personal health benefits to boost COVID-19 vaccination rates." Proceedings of the National Academy of Sciences 118(32).

Bartsch, Sarah M, Kelly J O'Shea, Marie C Ferguson, Maria Elena Bottazzi, Patrick T Wedlock, Ulrich Strych, James A McKinnell, Sheryl S Siegmund, Sarah N Cox, Peter J Hotez et al. 2020. "Vaccine efficacy needed for a COVID-19 coronavirus vaccine to prevent or stop an epidemic as the sole intervention." American journal of preventive medicine 59(4):493–503.

Bloniarz, Adam, Hanzhong Liu, Cun-Hui Zhang, Jasjeet S Sekhon and Bin Yu. 2016. "Lasso adjustments of treatment effect estimates in randomized experiments." Proceedings of the National Academy of Sciences 113(27):7383–7390.

Callaghan, Timothy, Ali Moghtaderi, Jennifer A Lueck, Peter J Hotez, Ulrich Strych, Avi Dor, Erika Franklin Fowler and Matt Motta. 2021. "Correlates and disparities of COVID-19 vaccine hesitancy." Social Science & Medicine .

Christensen, P Niels, Hank Rothgerber, Wendy Wood and David C Matz. 2004. "Social norms and identity relevance: A motivational approach to normative behavior." Personality and Social Psychology Bulletin 30(10):1295–1309.

Cialdini, Robert B. 2007. "Descriptive social norms as underappreciated sources of social control." Psychometrika 72(2):263–268.

Cialdini, Robert B, Carl A Kallgren and Raymond R Reno. 1991. A focus theory of normative conduct: A theoretical refinement and reevaluation of the role of norms in human behavior. In Advances in experimental social psychology. Vol. 24 Elsevier pp. 201–234.

Dai, Hengchen, Silvia Saccardo, Maria A Han, Lily Roh, Naveen Raja, Sitaram Vangala, Hardikkumar Modi, Shital Pandya, Michael Sloyan and Daniel M Croymans. 2021. "Behavioural nudges increase COVID-19 vaccinations." Nature 597(7876):404–409.

Druckman, James N and Thomas J Leeper. 2012. "Learning more from political communication experiments: Pretreatment and its effects." American Journal of Political Science 56(4):875–896.

Dubé, Eve, Dominique Gagnon, Noni E MacDonald et al. 2015. "Strategies intended to address vaccine hesitancy: Review of published reviews." Vaccine 33(34):4191–4203.

Fowler, Erika Franklin and Sarah E Gollust. 2015. "The content and effect of politicized health controversies." The ANNALS of the American Academy of Political and Social Science 658(1):155–171.

Fowler, Erika Franklin, Sarah E Gollust, Amanda F Dempsey, Paula M Lantz and Peter A Ubel. 2012. "Issue emergence, evolution of controversy, and implications for competitive framing: The case of the HPV vaccine." The International Journal of Press/Politics 17(2):169–189.

Franco, Annie, Neil Malhotra, Gabor Simonovits and LJ Zigerell. 2017. "Developing standards for post-hoc weighting in population-based survey experiments." Journal of Experimental Political Science 4(2):161–172.

Glenza, Jessica. 2021. "'There's a disconnect': after a rapid rollout why has US vaccine effort stalled?" Guardian, July 26, 2021. Downloaded July 28, 2021 from `https://www.theguardian.com/world/2021/jul/26/us-vaccine-effort-nuance-covid-coronavirus`.

Gravelle, Timothy B., Joseph B. Phillips, Jason Reifler and Thomas J. Scotto. In press. "Estimating the size of 'anti-vax' and vaccine hesitant populations in the US, UK, and Canada: Comparative latent class modelling of vaccine attitudes." Human Vaccines & Immunotherapeutics .

Hornsey, Matthew J, Josep Lobera and Celia Díaz-Catalán. 2020. "Vaccine hesitancy is strongly associated with distrust of conventional medicine, and only weakly associated with trust in alternative medicine." Social Science & Medicine 255:113019.

Jarrett, Caitlin, Rose Wilson, Maureen O'Leary, Elisabeth Eckersberger, Heidi J Larson et al. 2015. "Strategies for addressing vaccine hesitancy–A systematic review." Vaccine 33(34):4180–4190.

Ledford, Heidi, David Cyranoski and Richard Van Noorden. 2020. "The UK has approved a COVID vaccine-here's what scientists now want to know." Nature 588(7837):205–206.

Lee, Charlotte, Kathryn Whetten, Saad Omer, William Pan and Daniel Salmon. 2016. "Hurdles to herd immunity: Distrust of government and vaccine refusal in the US, 2002–2003." Vaccine 34(34):3972–3978.

MacDonald, Geoff and Mark R Leary. 2005. “Why does social exclusion hurt? The relationship between social and physical pain.” Psychological bulletin 131(2):202.

Milkman, Katherine L, Linnea Gandhi, Mitesh S Patel, Heather N Graci, Dena M Gromet, Hung Ho, Joseph S Kay, Timothy W Lee, Jake Rothschild, Jonathan E Bogard, Ilana Brody, Christopher F Chabris, Edward Chang, Gretchen B Chapman, Jennifer D Dannals, Noah J Goldstein, Amir Coren, Hal Hershfield, Alex Hirsch, Jillian Hmurovic, Samantha Horn, Dean S Karlan, Ariella S Kristal, cair Lamberton, Michelle N Meyer, Allison H Oakes, Maurice E Schweitzer, Maheen Shermohammed, Joachim Talloen, Caleb Warren, Ashley Whillans, Kuldeep N Yadav, Julian J Zlatev, Ron Berman, Chalanda N Evans, Rahul Ladhania, Jens Ludwig, Nina Mazar, Sendhil Mullainathan, Christopher K Snider, Jann Spiess, Eli Tsukayama, Lyle Ungar, Christophe Van den Bulte, Kevin G Volpp and Angela L Duckworth. N.d. “A 680,000-person megastudy of nudges to encourage vaccination in pharmacies.” Proceedings of the National Academy of Sciences. Forthcoming.

Miratrix, Luke W, Jasjeet S Sekhon, Alexander G Theodoridis and Luis F Campos. 2018. “Worth weighting? How to think about and use weights in survey experiments.” Political Analysis 26(3):275–291.

Nyhan, Brendan, Jason Reifler, Sean Richey and Gary L Freed. 2014. “Effective messages in vaccine promotion: a randomized trial.” Pediatrics 133(4):e835–e842.

Paluck, Elizabeth Levy. 2009. “Reducing intergroup prejudice and conflict using the media: a field experiment in Rwanda.” Journal of personality and social psychology 96(3):574.

Reid, Allecia E and Leona S Aiken. 2013. “Correcting injunctive norm misperceptions motivates behavior change: a randomized controlled sun protection intervention.” Health Psychology 32(5):551.

Reno, Raymond R, Robert B Cialdini and Carl A Kallgren. 1993. “The transsituational influence of social norms.” Journal of personality and social psychology 64(1):104.

Ryoo, Yuhosua and WooJin Kim. 2021. "Using descriptive and injunctive norms to encourage COVID-19 social distancing and vaccinations." Health Communication pp. 1–10.

Schultz, P Wesley, Jessica M Nolan, Robert B Cialdini, Noah J Goldstein and Vladas Griskevicius. 2007. "The constructive, destructive, and reconstructive power of social norms." Psychological science 18(5):429–434.

Sinclair, Samantha and Jens Agerström. 2021. "Do social norms influence young people's willingness to take the COVID-19 vaccine?" Health Communication pp. 1–8.

Smith-McLallen, Aaron and Martin Fishbein. 2008. "Predictors of intentions to perform six cancer-related behaviours: roles for injunctive and descriptive norms." Psychology, Health and Medicine 13(4):389–401.

Thaker, Jagadish and Somrita Ganchoudhuri. 2021. "The Role of Attitudes, Norms, and Efficacy on Shifting COVID-19 Vaccine Intentions: A Longitudinal Study of COVID-19 Vaccination Intentions in New Zealand." Vaccines 9(10):1132.

Wally, Christopher M and Linda D Cameron. 2017. "A randomized-controlled trial of social norm interventions to increase physical activity." Annals of Behavioral Medicine 51(5):642–651.
