# Supplementary material for: Minimal effects from injunctive norm and contentiousness treatments on COVID-19 vaccine intentions: evidence from 3 countries
Source: PNAS Nexus. 2022 May 13;1(2):pgac031. doi: 10.1093/pnasnexus/pgac031 (PMC9802041; doi:10.1093/pnasnexus/pgac031)
Supplement: pgac031_Supplemental_Files [file pgac031_supplemental_files.zip › PNASNEXUS-PNASNEXUS-2021-00156-s04.pdf]

Supplementary information: Additional results and estimation details

Contentiousness data and heterogeneous effects

The contention treatment provided respondents with Wave 2 data on how many people in Great Britain agree that whether to take an approved COVID-19 vaccine once available is a contentious issue there. Respondents answered on a five-point agree/disagree scale. In our heterogeneous effects analysis, we are thus unable to differentiate between people who overestimate and underestimate contention. We instead distinguish between respondents who previously agreed with this statement (somewhat or strongly) and those who did not.

Covariate selection

The eligible covariates for the covariate selection procedure were education, age group, gender, marital status, church attendance, region, party, ideology, living in a high incidence area, Cognitive Reflection Test (score) score, political knowledge, race, trust in health institutions, trust in the media, and pre-treatment vaccination intentions. In the New Hampshire (US) sample, region, CRT score, political knowledge, and trust in the media were not available as covariates. The pre-treatment vaccination intention question was administered in Wave 1 of the panel in the British sample, which was fielded September 11–29, 2020 — months before Wave 3 in which the present experiment was conducted (February 4–22, 2021). By contrast, the pre-treatment vaccination intention question was administered earlier in the same survey as the experiment in the Canadian and the New Hampshire (US) samples.[1]

[1]In the British and Canadian samples, the pre-treatment vaccination intention question mirrored the outcome measure. In the New Hampshire (US) sample, respondents answered a slightly different pre-treatment vaccine intention question. Codebooks for each survey are available at `https://osf.io/dp98m/?view_only=71747cc09b404fa6ae4f8a2340a6d269`.

In the British sample, the lasso selected trust in health institutions and Wave 2 vaccination intentions as predictors of Wave 3 vaccination intentions (as was being age 65 or older in a supplementary specification in which we code people who already received the vaccine as having a higher value on the outcome measure than those who said they were very likely to get it). In the Canadian sample, the lasso also selected trust in health institutions and pre-treatment vaccination intentions. In the New Hampshire sample from the US, the only covariate selected was pre-treatment vaccination intentions.

Balance tests

Table S1: Balance tests: New Hampshire sample (US)

	Control	Injunctive	Contention	*p*-value
University education	78%	77%	77%	p=.885
Age 18–34	9%	7%	7%	p=.454
Age 35–49	17%	16%	16%	p=.810
Age 50–64	36%	38%	41%	p=.146
Age 65+	38%	39%	35%	p=.347
Male	47%	49%	49%	p=.842
Married	70%	67%	72%	p=.697
Frequent church attendance	20%	22%	20%	p=.493
Republican	27%	26%	29%	p=.855
Democratic	61%	61%	60%	p=.526
Conservatism (three-point scale)	1.90	1.89	1.89	p=.914
High-incidence county	29%	30%	26%	p=.276
Non-white	4%	5%	5%	p=.820
Health trust (three-point scale)	2.63	2.57	2.63	p=.210
Pre-treatment vaccination intentions (four-point scale)	3.63	3.59	3.61	p=.713

Chi-squared tests were used for categorical variables; F-tests were used for ordinal variables. Pre-treatment vaccination intentions are coded as in the main text (treating already vaccinated as the same as respondents who indicate they are very likely to get vaccinated).

Table S2: Balance tests: Great Britain sample

	Control	Injunctive	Contention	*p*-value
University education	33%	31%	29%	p=.180
Age 18–34	15%	15%	14%	p=.866
Age 35–49	25%	24%	25%	p=.992
Age 50–64	31%	29%	31%	p=.424
Age 65+	29%	31%	30%	p=.405
Male	47%	46%	49%	p=.228
Married	49%	50%	48%	p=.593
Frequent church attendance	8%	8%	7%	p=.506
North East	3%	6%	6%	p=.010
North West	15%	16%	16%	p=.954
Yorkshire and the Humber	9%	7%	8%	p=.338
East Midlands	8%	8%	10%	p=.054
West Midlands	9%	11%	9%	p=.397
East of England	9%	9%	8%	p=.661
London	9%	9%	12%	p=.064
South East	14%	11%	11%	p=.077
South West	9%	9%	7%	p=.223
Wales	5%	4%	4%	p=.942
Scotland	10%	10%	8%	p=.249
Right PID	39%	41%	40%	p=.780
Left PID	47%	46%	45%	p=.769
Conservatism (11-point scale)	6.06	6.07	6.08	p=.976
High-incidence area	22%	22%	22%	p=.999
Political knowledge (six-point scale)	4.19	4.21	4.19	p=.874
Non-white	5%	5%	5%	p=.828
Political interest	3.04	2.99	3.00	p=.505
Health trust (four-point scale)	3.18	3.24	3.21	p=.081
Media trust (four-point scale)	2.24	2.27	2.27	p=.552
Pre-treatment vaccination intentions (six-point scale)	4.79	4.81	4.73	p=.497

Chi-squared tests were used for categorical variables; F-tests were used for ordinal variables.

Table S3: Balance tests: Canada

	Control	Injunctive	Contention	*p*-value
University education	28%	24	25%	p=.491
Age 18–34	27%	28%	27%	p=.872
Age 35–49	26%	25%	23%	p=.587
Age 50–64	26%	27%	29%	p=.747
Age 65+	20%	21%	21%	p=.940
Male	49%	49%	48%	p=.935
Married	53%	51%	53%	p=.906
Frequent church attendance	23%	23%	22%	p=.872
West	29%	32%	29%	p=.517
Ontario	41%	41%	35%	p=.128
Quebec	23%	22%	28%	p=.064
Atlantic	7%	5%	8%	p=.200
Lives in high-incidence area	27%	25%	27%	p=.921
Right PID	27%	25%	27%	p=.648
Left PID	56%	55%	53%	p=.668
Conservatism (11-point scale)	5.59	5.56	5.53	p=.927
Political knowledge (six-point scale)	2.91	3.01	2.87	p=.253
Non-white	19%	24%	22%	p=.201
Political interest (four-point scale)	3.26	3.17	3.11	p=.145
Health trust (four-point scale)	3.17	3.16	3.11	p=.457
Media trust (four-point scale)	2.69	2.62	2.59	p=.101
Pre-treatment vaccination intentions (six-point scale)	4.63	4.66	4.82	p=.238

Chi-squared tests were used for categorical variables; F-tests were used for ordinal variables. Pre-treatment vaccination intentions are coded as in the main text (treating already vaccinated as the same as respondents who indicate they are very likely to get vaccinated).

Statistical models including covariates

H1A

Table S4: Effect of injunctive norm/contention treatments on COVID vaccine intentions (GB)

	Coding of vaccinated respondents		
	"Very likely"	"Most likely"	Missing
Injunctive treatment	0.021	−0.020	−0.002
	(0.041)	(0.046)	(0.050)
Contention treatment	−0.009	−0.028	−0.015
	(0.044)	(0.049)	(0.053)
Health trust	1.060***	1.128***	1.362***
	(0.138)	(0.150)	(0.155)
Age 65+		0.606***	
		(0.039)	
Pre-treatment DV	1.884***	1.878***	2.114***
	(0.095)	(0.104)	(0.105)
Constant	3.333***	3.348***	2.875***
	(0.125)	(0.134)	(0.133)
N	3,186	3,186	2,498
R^2	0.344	0.357	0.395

OLS regression with robust standard errors in parentheses; ***p<0.005, **p<0.01; *p<0.05. Columns report results using different codings of responses from already-vaccinated participants ("very likely" results reported in main text).

Table S5: Effect of injunctive norm/contention treatments on COVID vaccine intentions (CA)

	Coding of vaccinated respondents		
	"Very likely"	"Most likely"	Missing
Injunctive treatment	0.074	0.071	0.090
	(0.050)	(0.051)	(0.050)
Contention treatment	−0.005	−0.011	0.010
	(0.053)	(0.054)	(0.052)
Health trust	0.623***	0.646***	0.558***
	(0.160)	(0.169)	(0.150)
Pre-treatment DV	0.887***	0.882***	0.895***
	(0.019)	(0.020)	(0.018)
Constant	0.019	0.030	0.023
	(0.103)	(0.103)	(0.101)
N	1,309	1,309	1,294
R^2	0.812	0.807	0.821

OLS regression with robust standard errors in parentheses; ***p<0.005, **p<0.01; *p<0.05. Columns report results using different codings of responses from already-vaccinated participants ("very likely" results reported in main text).

Table S6: Effect of injunctive norm/contention treatments on COVID vaccine intentions (NH [US])

	Coding of vaccinated respondents		
	"Very likely"	"Most likely"	Missing
Injunctive treatment	0.015	0.007	0.018
	(0.019)	(0.021)	(0.021)
Contention treatment	−0.007	−0.012	−0.007
	(0.023)	(0.025)	(0.025)
Pre-treatment DV	1.629***	1.538***	1.631***
	(0.013)	(0.014)	(0.013)
Constant	1.143***	1.332***	1.141***
	(0.045)	(0.047)	(0.046)
N	1,969	1,969	1,775
R^2	0.935	0.927	0.934

OLS regression with robust standard errors in parentheses; ***p<0.005, **p<0.01; *p<0.05. Columns report results using different codings of responses from already-vaccinated participants ("very likely" results reported in main text).

H1B

Table S7: Effect of injunctive norm/contention treatments by prior norm estimation (GB)

	Coding of vaccinated respondents		
	"Very likely"	"Most likely"	Missing
Injunctive treatment	0.045	0.016	0.034
	(0.044)	(0.050)	(0.055)
Underestimated norms	-0.418^{***}	-0.382^{***}	-0.466^{***}
	(0.072)	(0.078)	(0.081)
Overestimated norms	0.129^{**}	0.156^{**}	0.114
	(0.048)	(0.057)	(0.062)
Injunctive × underestimated norms	−0.086	−0.107	−0.048
	(0.117)	(0.125)	(0.128)
Injunctive × overestimated norms	−0.022	−0.034	−0.067
	(0.071)	(0.086)	(0.088)
Contention treatment	−0.014	−0.030	−0.024
	(0.043)	(0.048)	(0.052)
Health trust	0.959^{***}	1.028^{***}	1.236^{***}
	(0.133)	(0.146)	(0.153)
Age 65+		0.561^{***}	
		(0.037)	
Pre-treatment DV	1.681^{***}	1.695^{***}	1.886^{***}
	(0.095)	(0.103)	(0.105)
Constant	3.641^{***}	3.638^{***}	3.237^{***}
	(0.128)	(0.138)	(0.140)
N	2,982	2,982	2,326
R^2	0.361	0.371	0.407

OLS regression with robust standard errors in parentheses; $^{***}p<0.005$, $^{**}p<0.01$; $^{*}p<0.05$. Columns report results using different codings of responses from already-vaccinated participants ("very likely" results reported in main text).

Table S8: Effect of injunctive norm/contention treatments by prior norm estimation (CA)

	Coding of vaccinated respondents		
	"Very likely"	"Most likely"	Missing
Injunctive treatment	0.042	0.036	0.060
	(0.070)	(0.071)	(0.069)
Underestimated norms	-0.232^{*}	-0.242^{*}	-0.202^{*}
	(0.095)	(0.097)	(0.092)
Overestimated norms	0.050	0.059	0.038
	(0.054)	(0.055)	(0.054)
Injunctive × underestimated	0.185	0.186	0.184
	(0.136)	(0.140)	(0.132)
Injunctive × overestimated	−0.023	−0.018	−0.029
	(0.081)	(0.083)	(0.081)
Contention treatment	0.001	−0.005	0.016
	(0.052)	(0.053)	(0.052)
Health trust	0.570^{***}	0.587^{***}	0.518^{***}
	(0.155)	(0.161)	(0.147)
Pre-treatment DV	0.869^{***}	0.862^{***}	0.880^{***}
	(0.022)	(0.024)	(0.021)
Constant	0.176	0.198	0.152
	(0.127)	(0.129)	(0.127)
N	1,307	1,307	1,292
R^2	0.814	0.809	0.823

OLS regression with robust standard errors in parentheses; $^{***}p<0.005$, $^{**}p<0.01$; $^{*}p<0.05$. Columns report results using different codings of responses from already-vaccinated participants ("very likely" results reported in main text).

RQ1

Table S9: Effect of injunctive norm/contention treatments by prior contention estimation (GB)

	Coding of vaccinated respondents		
	"Very likely"	"Most likely"	Missing
Contention treatment	−0.036	−0.051	−0.017
	(0.060)	(0.066)	(0.077)
Contention accuracy	−0.004	−0.005	0.007
	(0.042)	(0.046)	(0.050)
Contention × accuracy	0.045	0.040	0.003
	(0.074)	(0.083)	(0.092)
Injunctive treatment	0.021	−0.020	−0.002
	(0.041)	(0.046)	(0.050)
Health trust	1.059***	1.127***	1.362***
	(0.138)	(0.150)	(0.117)
Age 65+		0.606***	
		(0.039)	
Pre-treatment DV	1.886***	1.880***	2.116***
	(0.096)	(0.104)	(0.070)
Constant	3.334***	3.350***	2.869***
	(0.130)	(0.139)	(0.094)
N	3,185	3,185	2,497
R^2	0.344	0.357	0.395

OLS regression with robust standard errors in parentheses; ***p<0.005, **p<0.01; *p<0.05. Columns report results using different codings of responses from already-vaccinated participants ("very likely" results reported in main text).

RQ2

Table S10: Effect of injunctive norm/contention treatments by fact-check treatment status (GB)

	Coding of vaccinated respondents		
	"Very likely"	"Most likely"	Missing
Injunctive treatment	0.045	−0.050	0.031
	(0.076)	(0.084)	(0.095)
Contention treatment	0.016	−0.026	0.020
	(0.079)	(0.089)	(0.100)
Wave 2 fact-check treatment	0.026	−0.042	0.056
	(0.063)	(0.070)	(0.078)
Wave 3 fact-check treatment	0.016	0.013	0.019
	(0.063)	(0.070)	(0.077)
Injunctive treatment × Wave 2 fact-check treatment	−0.048	0.043	−0.068
	(0.083)	(0.092)	(0.102)
Injunctive treatment × Wave 3 fact-check treatment	0.001	0.017	0.006
	(0.083)	(0.092)	(0.101)
Contention treatment × Wave 2 fact-check treatment	−0.047	0.029	−0.047
	(0.088)	(0.099)	(0.108)
Contention treatment × Wave 3 fact-check treatment	−0.003	−0.032	−0.019
	(0.088)	(0.098)	(0.107)
Health Trust	1.062***	1.126***	1.365***
	(0.138)	(0.150)	(0.155)
Age 65+		0.606***	
		(0.039)	
Pre-Treatment DV	0.377***	0.376***	0.422***
	(0.019)	(0.021)	(0.021)
Constant	2.935***	2.988***	2.413***
	(0.144)	(0.155)	(0.155)
Observations	3,186	3,186	2,498
R^2	0.344	0.357	0.395
Joint F-Test	0.104	0.135	0.120

OLS regression with robust standard errors in parentheses; ***p<0.005, **p<0.01; *p<0.05. Columns report results using different codings of responses from already-vaccinated participants ("very likely" results reported in main text).

Table S11: Effect of injunctive norm/contention treatments by fact-check treatment status (CA)

	Coding of vaccinated respondents		
	"Very likely"	"Most likely"	Missing
Injunctive treatment	0.073	0.076	0.099
	(0.077)	(0.076)	(0.075)
Contention treatment	−0.030	−0.026	−0.006
	(0.080)	(0.079)	(0.078)
Fact-check treatment	0.021	0.018	0.011
	(0.077)	(0.076)	(0.076)
Injunctive treatment × fact-check treatment	−0.005	−0.004	−0.019
	(0.102)	(0.100)	(0.099)
Contention treatment × fact-check treatment	0.040	0.043	0.034
	(0.109)	(0.107)	(0.105)
Health trust	0.642***	0.619***	0.556***
	(0.167)	(0.159)	(0.149)
Pre-Treatment DV	0.882***	0.887***	0.895***
	(0.020)	(0.019)	(0.018)
Constant	0.022	0.012	0.019
	(0.117)	(0.116)	(0.115)
Observations	1,309	1,309	1,294
R^2	0.807	0.812	0.821
Joint F-Test	0.104	0.135	0.120

OLS regression with robust standard errors in parentheses; $^{***}p<0.005$, $^{**}p<0.01$; $^{*}p<0.05$. Columns report results using different codings of responses from already-vaccinated participants ("very likely" results reported in main text).

Statistical models excluding covariates

Table S12: Effect of injunctive norm/contention treatments on COVID vaccine intentions (GB)

	Coding of vaccinated respondents		
	"Very likely"	"Most likely"	Missing
Injunctive treatment	0.048	0.026	0.078
	(0.050)	(0.057)	(0.063)
Contention treatment	−0.025	−0.036	−0.022
	(0.056)	(0.062)	(0.070)
Constant	5.532^{***}	5.760^{***}	5.393^{***}
	(0.038)	(0.043)	(0.049)
N	3,190	3,190	2,502
R^2	0.001	0.0004	0.001

OLS regression with robust standard errors in parentheses; $^{***}p<0.005$, $^{**}p<0.01$; $^{*}p<0.05$. Columns report results using different codings of responses from already-vaccinated participants ("very likely" results reported in main text).

Table S13: Effect of injunctive norm/contention treatments on COVID vaccine intentions (CA)

	Coding of vaccinated respondents		
	"Very likely"	"Most likely"	Missing
Injunctive treatment	0.098	0.101	0.095
	(0.115)	(0.115)	(0.115)
Contention treatment	0.149	0.145	0.155
	(0.123)	(0.123)	(0.123)
Constant	4.580^{***}	4.589^{***}	4.567^{***}
	(0.086)	(0.086)	(0.086)
N	1,315	1,315	1,303
R^2	0.001	0.001	0.001

OLS regression with robust standard errors in parentheses; $^{***}p<0.005$, $^{**}p<0.01$; $^{*}p<0.05$. Columns report results using different codings of responses from already-vaccinated participants ("very likely" results reported in main text).

H1A

Table S14: Effect of injunctive norm/contention treatments on COVID vaccine intentions (NH [US])

	Coding of vaccinated respondents		
	"Very likely"	"Most likely"	Missing
Injunctive treatment	−0.041	−0.033	−0.053
	(0.078)	(0.082)	(0.086)
Contention treatment	−0.050	−0.045	−0.060
	(0.083)	(0.087)	(0.090)
Constant	5.389***	5.480***	5.328***
	(0.057)	(0.059)	(0.062)
N	2,018	2,018	1,824
R^2	0.0002	0.0001	0.0003

OLS regression with robust standard errors in parentheses; ***p<0.005, **p<0.01; *p<0.05. Columns report results using different codings of responses from already-vaccinated participants ("very likely" results reported in main text).

H1B

Table S15: Effect of injunctive norm/contention treatments by prior norm estimation (GB)

	Coding of vaccinated respondents		
	"Very likely"	"Most likely"	Missing
Injunctive treatment	0.104*	0.097	0.137*
	(0.042)	(0.049)	(0.054)
Underestimated norms	−0.685***	−0.755***	−0.779***
	(0.094)	(0.103)	(0.109)
Overestimated norms	0.189***	0.243***	0.232***
	(0.050)	(0.061)	(0.067)
Injunctive × underestimated norms	−0.142	−0.171	−0.132
	(0.149)	(0.161)	(0.169)
Injunctive × overestimated norms	−0.047	−0.062	−0.055
	(0.067)	(0.085)	(0.089)
Constant	5.664***	5.894***	5.564***
	(0.030)	(0.034)	(0.038)
N	2,985	2,985	2,329
R^2	0.079	0.078	0.087

OLS regression with robust standard errors in parentheses; ***p<0.005, **p<0.01; *p<0.05. Columns report results using different codings of responses from already-vaccinated participants ("very likely" results reported in main text).

Table S16: Effect of injunctive norm/contention treatments by prior norm estimation (CA)

	Coding of vaccinated respondents		
	"Very likely"	"Most likely"	Missing
Injunctive treatment	0.014	0.008	0.022
	(0.147)	(0.147)	(0.147)
Underestimated norms	-1.644^{***}	-1.650^{***}	-1.636^{***}
	(0.165)	(0.165)	(0.165)
Overestimated norms	0.504^{***}	0.510^{***}	0.504^{***}
	(0.114)	(0.115)	(0.115)
Injunctive × underestimated	0.178	0.193	0.149
	(0.261)	(0.262)	(0.261)
Injunctive × overestimated	−0.083	−0.064	−0.101
	(0.186)	(0.188)	(0.188)
Constant	4.846^{***}	4.852^{***}	4.838^{***}
	(0.091)	(0.091)	(0.091)
N	1,312	1,312	1,300
R^2	0.216	0.216	0.215

OLS regression with robust standard errors in parentheses; $^{***}p<0.005$, $^{**}p<0.01$; $^{*}p<0.05$. Columns report results using different codings of responses from already-vaccinated participants ("very likely" results reported in main text).

RQ1

Table S17: Effect of injunctive norm/contention treatments by prior contention perception (GB)

	Coding of vaccinated respondents		
	"Very likely"	"Most likely"	Missing
Contention treatment	−0.048	−0.027	−0.078
	(0.071)	(0.080)	(0.090)
Contention accuracy	−0.084	−0.089	−0.104
	(0.050)	(0.056)	(0.062)
Contention × accuracy	−0.006	−0.040	0.020
	(0.095)	(0.106)	(0.119)
Constant	5.610^{***}	5.828^{***}	5.501^{***}
	(0.037)	(0.042)	(0.046)
N	3,189	3,189	2,501
R^2	0.002	0.002	0.002

OLS regression with robust standard errors in parentheses; $^{***}p<0.005$, $^{**}p<0.01$; $^{*}p<0.05$. Columns report results using different codings of responses from already-vaccinated participants ("very likely" results reported in main text).

RQ2

Table S18: Effect of injunctive norm/contention treatments by fact-check treatment status (GB)

	Coding of vaccinated respondents		
	"Very likely"	"Most likely"	Missing
Injunctive treatment	0.103	0.027	0.199
	(0.092)	(0.104)	(0.119)
Contention treatment	0.055	−0.003	0.124
	(0.102)	(0.115)	(0.132)
Wave 2 fact-check treatment	0.064	−0.025	0.156
	(0.078)	(0.088)	(0.100)
Wave 3 fact-check treatment	0.058	0.041	0.092
	(0.077)	(0.087)	(0.098)
Injunctive treatment × Wave 2 fact-check treatment	−0.074	0.033	−0.181
	(0.101)	(0.114)	(0.128)
Injunctive treatment × Wave 3 fact-check treatment	−0.035	−0.036	−0.050
	(0.101)	(0.113)	(0.127)
Contention treatment × Wave 2 fact-check treatment	−0.115	−0.013	−0.231
	(0.112)	(0.125)	(0.141)
Contention treatment × Wave 3 fact-check treatment	−0.043	−0.054	−0.050
	(0.112)	(0.125)	(0.140)
Constant	5.470***	5.753***	5.263***
	(0.074)	(0.085)	(0.099)
Observations	3,190	3,190	2,502
R^2	0.001	0.001	0.003
Joint F-Test	0.264	0.098	0.633

OLS regression with robust standard errors in parentheses; ***p<0.005, **p<0.01; *p<0.05. Columns report results using different codings of responses from already-vaccinated participants ("very likely" results reported in main text).

Table S19: Effect of injunctive norm/contention treatments by fact-check treatment status (CA)

	Coding of vaccinated respondents		
	"Very likely"	"Most likely"	Missing
Injunctive treatment	0.081	0.078	0.075
	(0.163)	(0.162)	(0.163)
Contention treatment	0.068	0.077	0.089
	(0.175)	(0.174)	(0.175)
Fact-check treatment	0.005	0.014	0.026
	(0.173)	(0.172)	(0.173)
Injunctive treatment × fact-check treatment	0.038	0.038	0.038
	(0.231)	(0.229)	(0.231)
Contention treatment × fact-check treatment	0.162	0.153	0.141
	(0.246)	(0.245)	(0.247)
Constant	4.587***	4.573***	4.554***
	(0.120)	(0.119)	(0.121)
Observations	1,315	1,315	1,303
R^2	0.002	0.002	0.002
Joint F-Test	0.343	0.329	0.367

OLS regression with robust standard errors in parentheses; ***p<0.005, **p<0.01; *p<0.05. Columns report results using different codings of responses from already-vaccinated participants ("very likely" results reported in main text).

Robustness check

To verify the robustness of the findings reported in the main text, we also conducted additional exploratory analyses testing if the injunctive norm treatment had a statistically significant effect at any level of perceived injunctive norms (measured in a previous wave of the UK sample and immediately before the experiment in the Canadian sample). We estimated interactions between the norm treatment and perceived injunctive norms in each sample using a kernel estimator in the `interflex` package in R, which allows for non-linear interactions (Hainmueller, Mummolo and Xu 2019). The results are in Figure S1 below.

In the British sample, the null effect of the injunctive norm treatment is consistent across the entire range of perceived norms. In the Canadian sample, only extreme under-estimators respond positively to the injunctive norm treatment — specifically, those who think 12% or fewer Canadians would want them to get a vaccine, which amounts to 27 respondents or just 2% of the sample.

Figure S1: Treatment effect estimates on vaccine intention by previously perceived injunctive norm

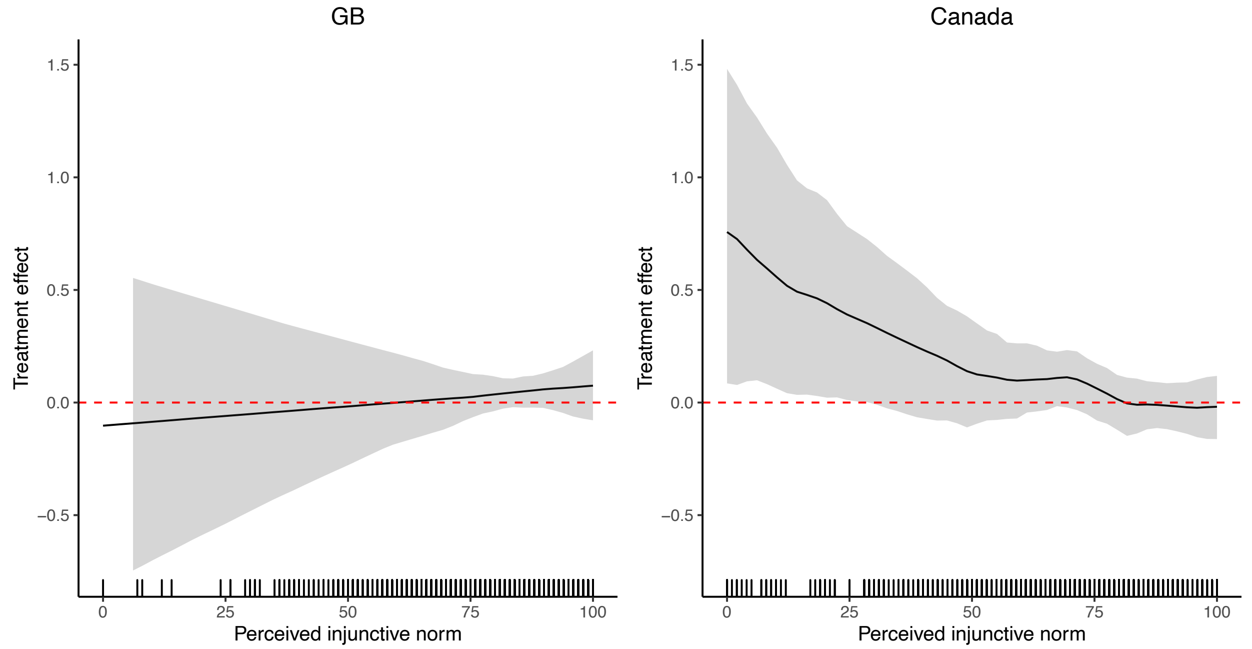

Marginal effect estimates of treatment assignment on vaccine intention by perceived levels of the injunctive norm in favor of COVID-19 vaccination. Estimates created using kernel estimator in the `interflex` package in R, which allows for non-linear interactions (Hainmueller, Mummolo and Xu 2019).
